# Experiences of healthcare professionals providing palliative care in home settings - a scoping review

**DOI:** 10.1186/s12904-025-01728-z

**Published:** 2025-03-28

**Authors:** Luke Tan, Sonia Sheri, Yun Yao Goh, Raeanne Fong, Ranitha Govindasamy, Yun Ting Ong, Nur Amira Binte Abdul Hamid, Tessa Li Xiang Low, Lalit Kumar Radha Krishna

**Affiliations:** 1 Yong Loo Lin School of Medicine, National University of Singapore, NUHS Tower Block, Level 11, Block 1E, Kent Ridge Road, Singapore, 119228 Singapore; 2 Lee Kong Chian School of Medicine, Nanyang Technological University, 11 Mandalay Road, Singapore, 308207 Singapore; 3 Centre for Biomedical Ethics, National University of Singapore, Block MD11, 10 Medical Drive, Singapore, #02-03, 117597 Singapore; 4 Division of Cancer Education, National Cancer Centre Singapore, 30 Hospital Boulevard, Singapore, 168583 Singapore; 5 Division of Supportive and Palliative Care, National Cancer Centre Singapore, 30 Hospital Boulevard, Singapore, 168583 Singapore; 6 Department of Psychological Medicine, Yong Loo Lin School of Medicine, University Medicine Cluster, National University of Singapore, MD1 Tahir Foundation Building, 12 Science Drive 2, Singapore, 117549 Singapore; 7 Duke-NUS Medical School, National University of Singapore, 8 College Road, Singapore, 169857 Singapore; 8 Palliative Care Institute Liverpool, Academic Palliative & End of Life Care Centre, Cancer Research Centre, University of Liverpool, 200 London Road, Liverpool, L3 9TA UK; 9 Health Data Science, University of Liverpool, Whelan Building, The Quadrangle, Brownlow Hill, Liverpool, L69 3GB UK; 10 PalC, The Palliative Care Centre for Excellence in Research and Education, Dover Park Hospice, 10 Jalan Tan Tock Seng, Singapore, 308436 Singapore

**Keywords:** Homecare, Palliative care, Healthcare professionals, Personhood, Costs of caring, Compassion fatigue

## Abstract

**Background:**

The growing preference for home-based end-of-life care accords a dignified death for terminally ill patients. However, for healthcare professionals (HCPs) involved, this caregiving approach is embedded with unique psychosocial, practical and emotional stressors. Without sufficient support, HCPs face higher risks of moral distress, compassion fatigue, vicarious trauma, secondary traumatic stress and burnout—collectively known as the costs of caring—that precipitate depersonalisation and compromised patient care. Despite its far-reaching implications, current understanding of the costs of caring amongst HCPs in home-based settings remains remiss. Thus, we conduct a scoping review to investigate the experiences of HPCs providing home-based palliative care to terminally ill adult oncology patients.

**Methods:**

Outlined by the Systematic Evidence-Based Approach and PRISMA guidelines, searches for relevant articles published between 1st January 2000 and 1st October 2024 were performed on PubMed, Embase, Scopus, PsycINFO and CINAHL databases. Selected articles underwent concurrent and independent thematic and content analyses. Central themes and categories were extracted and merged, forming key domains that framed the discussion.

**Results:**

Of 5676 titles and abstracts screened, 543 full-text articles were reviewed. 20 full-text articles were analysed for inclusion. Four key domains emerged: (1) motivations to practice palliative care; (2) impact on personhood of HCPs (3) challenges faced by HCPs; and (4) support systems for HCPs.

**Conclusion:**

Providing home-based palliative care to adult oncology patients is fulfilling for HCPs—fostering meaningful professional relationships with patients, a more holistic perspective of life and death and a heightened sense of personal accomplishment. However, HCPs may encounter dissonance amidst conflicts between their dominant beliefs and new experiences, leading to burnout, depersonalisation and poor care delivery that are further exacerbated by the costs of caring, if inadequately addressed. Longitudinal and accessible personalised and organisational support is key to sustaining HCPs’ capacity to deliver compassionate and high-quality palliative care. The use of the Ring Theory of Personhood framework in this review provides an avenue for the structuring of such support systems.

**Supplementary Information:**

The online version contains supplementary material available at 10.1186/s12904-025-01728-z.

## Background

An increasing number of Singaporeans are choosing to live their final days at home [1–7]. This is reflected in the expansion of Singapore’s palliative homecare system that aims to increase its capacity by 50% to meet the needs of a ‘superaged’ population [8]. This preference for palliative homecare in Singapore is also deeply entwined with the desire to maintain dignity and comfort in a familiar environment [1–7], buttressed by dominant Asian values on family cohesion and filial piety as patients spend their final days surrounded by loved ones [9]. However, providing care for dying patients in their homes brings psychosocial, practical and emotional stressors to familial and non-familial caregivers [10–15] that are often compounded by inadequate support [16–20].

Recent studies have revealed that there is a cost of caring experienced by healthcare professionals (HCPs), defined as trained providers of health services such as physicians, nurses and allied health professionals, as they care for end-of-life patients. This cost of caring extends beyond traditional notions of compassion fatigue and is defined by a complex and dynamic web of moral distress, compassion fatigue, vicarious trauma and secondary traumatic stress [21–32] as personal and professional boundaries blur [33]. If HCPs are left unsupported [16–20], the costs of caring may culminate in burnout [21, 34–46], leading to depersonalisation, derealisation and compromised patient care [23–27]. It is proposed that HCPs in homecare services face an increased risk of the costs of caring. For one, HCPs practicing in palliative homecare lack consistent access to the resources in a tertiary setting, including larger palliative care departments, support from other specialties and professions and specialty palliative care training [8, 47]. Furthermore, compared to familial caregivers, HCPs may have less awareness of patients’ preferences and values—limiting their ability to provide effective holistic care to alleviate suffering [47] which, in itself, is a source of moral distress.

Given that the costs of caring amongst HCPs practicing home palliative care [48–50] remain poorly delineated and have yet to be studied from a whole-person holistic lens of personhood [51], this study aims to illuminate the experiences of HCPs providing home-based care for terminally ill adult oncology patients. The decision was made to focus on adult oncology patients as they constitute a significant proportion of individuals receiving palliative care [8, 52–56]. While patients with life-limiting non-cancer illnesses, such as progressive neurological conditions, also benefit from palliative care [8], their clinical, spiritual, social and ethical needs are diverse and unique. Addressing these distinct aspects warrants separate studies to ensure comprehensive understanding and tailored care approaches.

Thus, the research question for this scoping review is, ‘*What are the experiences of healthcare professionals caring for adult oncology patients receiving palliative care at home?*’ to lay the foundation for extending better support to HCPs.

## Methods

We utilise the Systematic Evidence-Based Approach (SEBA) methodology and the Ring Theory of Personhood (RToP) theoretical framework to guide our review.

### The systematic evidence-based approach

**Fig. 1:**
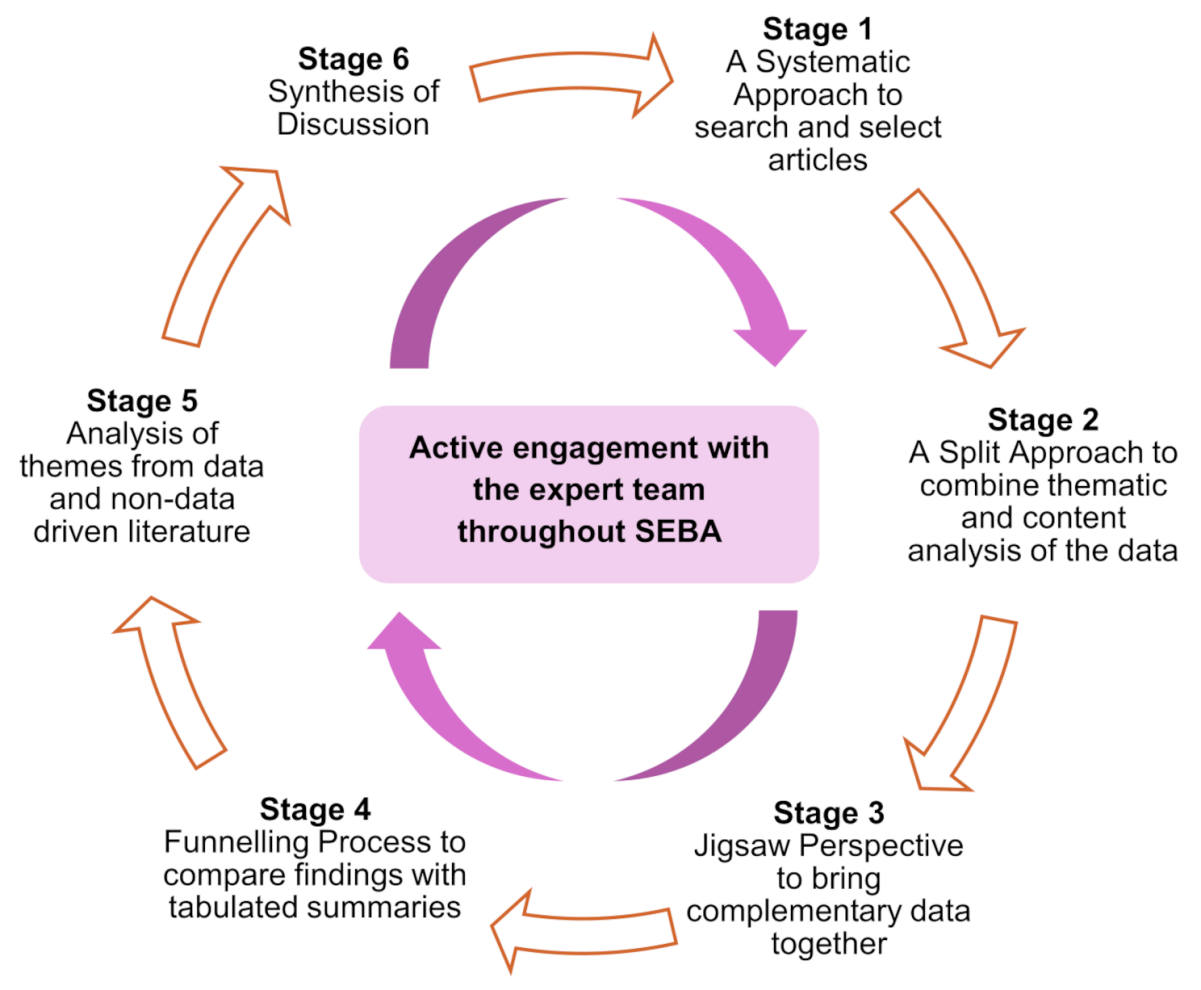
Stages of the systematic evidence-based approach

The six-staged SEBA methodology instils consistency, reproducibility and trustworthiness as a methodological framework to search, collect and analyse current literature for scoping reviews (Fig. 1). The SEBA methodology adopts a constructivist approach [57–63] and relativist lens [64–69] to develop a holistic and nuanced understanding of the costs of caring as a sociocultural construct [41–44].

### The ring theory of personhood

**Fig. 2:**
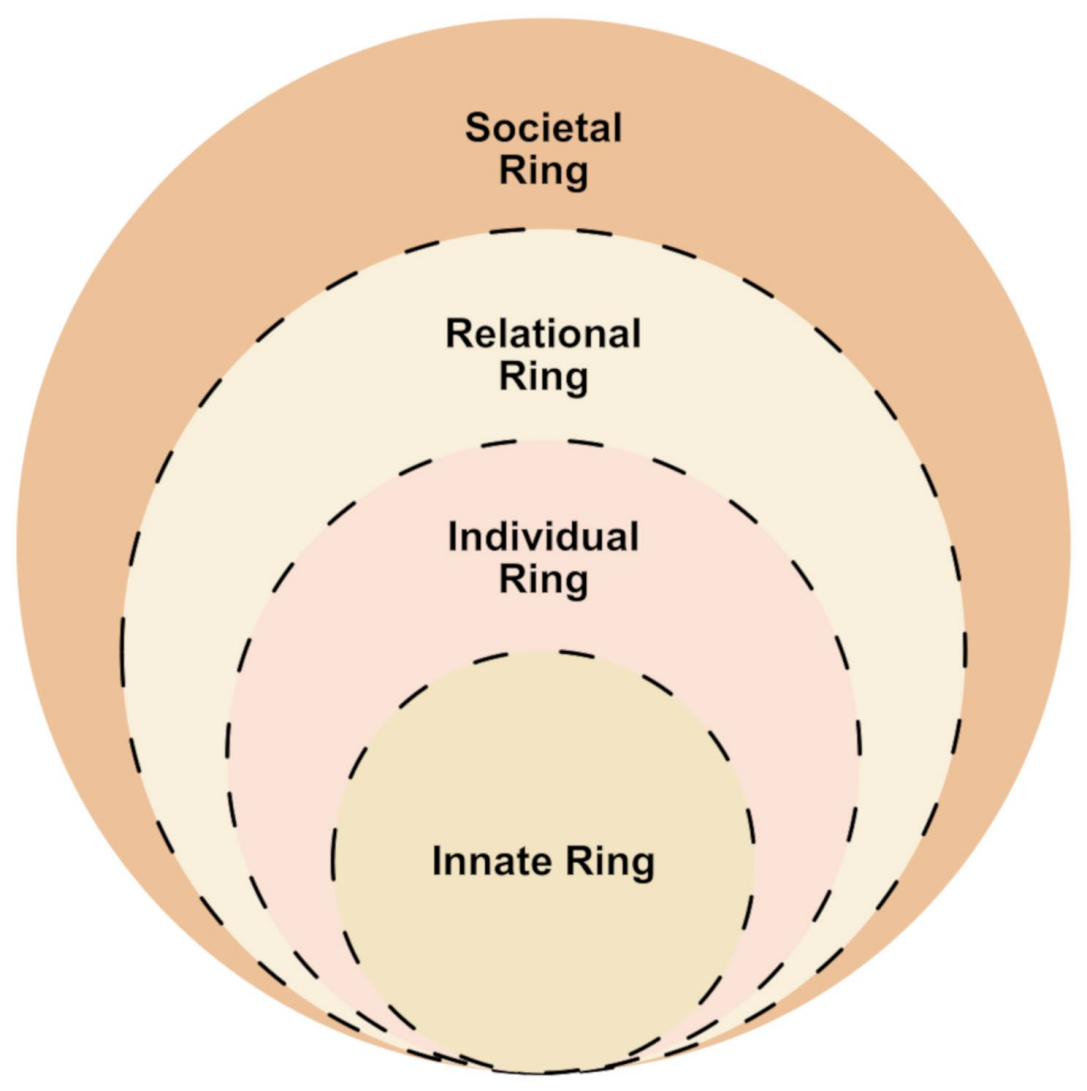
The ring theory of personhood

The RToP is a clinically evidenced theoretical framework that captures a HCP’s self-concept of personhood (‘what makes you, you’), sketching their evolving belief systems by recording changes in one or more domains of personhood (Fig. 2). The RToP has been used to map the impacts of *resonance* (which occurs when a HCP’s experiences, reflections and meaning-making align with their dominant beliefs) and *dissonance* (which occurs when such experiences conflict, rather than align, with existing beliefs) on different elements of personhood and resultingly, their belief systems and identity. The RToP also accounts for the effects of growing competencies, deeper insights and guided reflections [70–72] drawn from clinical experiences [24, 73] that impact personhood, identity and belief systems.

The RToP posits that belief systems comprise four intertwined Innate, Individual, Relational and Societal Rings. The Innate Ring contains belief systems informed by the HCP’s demographic features and spiritual and religious beliefs. The belief systems in the Individual Ring draw on the HCP’s conscious function, including their behaviour, emotions and personality. Close familial relationships and friendships drive belief systems housed in the Relational Ring whilst sociocultural, professional, legal and ethical norms, rights, expectations, roles and responsibilities draw on the belief systems in the Societal Ring.

### Stage 1 of SEBA: the systematic approach

Overseeing the research process was an expert team of medical librarians from the Yong Loo Lin School of Medicine (YLLSOM), along with local educational experts and clinicians at the National Cancer Centre Singapore, the Palliative Care Institute Liverpool and YLLSOM. Their involvement served to bolster the reliability and transparency of the methodology, ensuring compliance with the PRISMA-ScR guidelines (see Additional File 1).

#### Determining the research question and inclusion criteria

Steered by Population, Comparison and Context (PCC) framework of inclusion and exclusion (Table 1), the primary research question was determined as follows: ‘*What are the experiences of healthcare professionals caring for adult oncology patients receiving palliative care at home?*’. This was complemented by the following secondary research questions: ‘*What factors impact a healthcare professional’s experiences caring for adult oncology patients receiving palliative care at home?*’ and ‘*How are healthcare professionals impacted by caring for adult oncology patients receiving palliative care at home?*’.

**Table 1:** Population, comparison and context (PCC) framework and inclusion and exclusion criteria applied to database search

	Inclusion	Exclusion
Population	- Healthcare professionals providing palliative care for adult oncology patients at home	- Caregivers who are not healthcare professionals - Caregivers caring for terminally ill patients in an institutional setting - Caregivers who care for paediatric and/or non-oncology patients - Caregivers who care for patients with unspecified illnesses
Concept	- Focused on experiences, perceptions and needs of caregivers	NA
Context	- Articles about homecare setting including home hospice - Published from 1 Jan 2000 to 1 Oct 2024	- Articles that fall outside of date range - Care of patients out of homecare setting (hospice) - Non-English publications

#### Searching

Searches for articles published between 1st January 2000 and 1st October 2024 on PubMed, Embase, Scopus, PsycINFO and CINAHL databases commenced between 1st October 2024 and 2nd October 2024. ‘Snowballing’ through review of the references of included articles identified additional articles. Additional File 2 details the full search strategy for the database searches.

#### Extracting and charting

The titles and abstracts were independently reviewed by members of the research team, who then discussed and deconflicted their findings through Sandelowski and Barroso’s [74] process of “*negotiated consensual validation*” wherein “*research team members articulate*,* defend*,* and persuade others of the ‘cogency’ or ‘incisiveness’ of their points of view*”. Subsequently, the full text of each shortlisted abstract was reviewed. Finalised articles were summarised to retain key information and underwent quality appraisal (see Additional File 3).

### Stage 2 of SEBA: split approach

The data from the searches were concurrently analysed by two research teams (LT, SS, YYG, RF, RG, YTO, LKRK). One team utilised Braun and Clarke’s [75] approach to thematic analysis, synthesising codes from the ‘surface’ meaning of the included articles to arrive at semantic themes. The other team employed Hsieh and Shannon’s [76] directed content analysis, utilising predetermined codes drawn from Kaup et al.’s [77] qualitative study titled, ‘*Care for Dying Patients at Midlife*’, which surveyed nurses’ experiences of caring for midlife patients in specialised palliative homecare. Consensus on the key themes and categories of each article was attained through “*negotiated consensual validation*” [74].

### Stage 3 of SEBA: the jigsaw perspective and stage 4 of SEBA: the funnelling process

As complementary qualitative data gives “*a richer*,* more nuanced understanding of a given phenomenon*” [78], overlaps in themes and categories were combined and then compared with the summaries of the included articles to ensure that the ‘jigsaw pieces’ appropriately represented the data across the included articles.

### Stage 5 of SEBA: analysis of evidence-based and non-data driven literature

The inclusion of non-peer-reviewed or non-evidence-based literature raised concerns about potential bias in data analysis. However, by comparing themes/categories from such literature with that of peer-reviewed data, it was ascertained that the research data remained unbiased despite the inclusion of non-peer-reviewed or non-evidence-based literature.

## Results

**Fig. 3:**
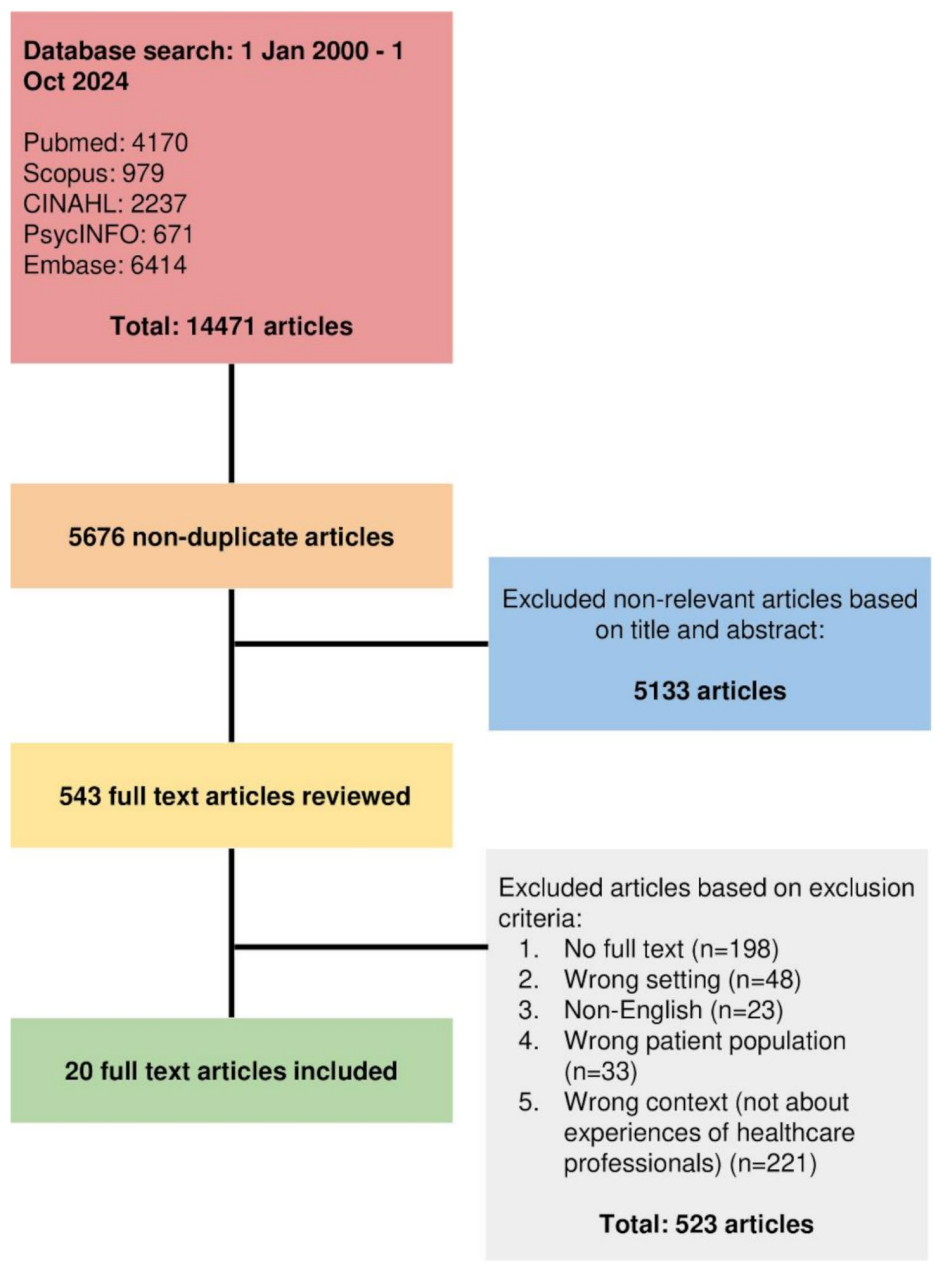
PRISMA flowchart

In total, 5676 titles and abstracts were identified after removal of duplicates. 543 full-text articles were then reviewed and 20 full-text articles were analysed (Fig. 3).

Among the 20 articles analysed, nurses were the most frequently studied healthcare professionals (*n* = 17), followed by general practitioners (GPs) (*n* = 9). Community health workers (CHWs), physiotherapists, psychologists and occupational therapists were each represented in a single study (*n* = 1).

Four key domains were identified: (1) motivations to practice palliative care; (2) impact on personhood of HCPs; (3) challenges faced by HCPs; and (4) support systems for HCPs.

### Domain 1: motivations to practice palliative care

Providing home-based palliative care for adult oncology patients is meaningful and rewarding for HCPs across different professions (e.g. physicians, nurses, CHWs) [30, 79–82]. Griffiths et al.’s [79] interviews with homecare nurses in the United Kingdom found that they attached importance to their role, believing they had “*a lot to offer*”, were practicing “*real nursing*” and “*making a difference to somebody’s life*”. This notion was also echoed in Potts et al.’s [30] interview study with informal and, at times, unlicensed rural medical practitioners who served as CHWs. The study revealed that CHWs were highly valued by patients and their families as companions providing clinical, psycho-emotional and spiritual support despite their varied training in healthcare. CHWs found that this recognition by the patients and their families, as well as the ability to alleviate suffering holistically, made their work rewarding [30].

### Domain 2: impact on personhood of HCPs

Many HCPs, especially those who report feeling supported at work, find their work gratifying and are invigorated by maintaining an open and emotional climate with patients and families [77] and enhancing healthcare-community ties [83]. HCPs in the homecare setting also report being privileged by the opportunity to build deeper relationships with patients and their families over time [30, 79, 83, 84]. This allows them to better understand and cater to the unique needs of patients [30, 83].

However, when unsupported at an institutional level, HCPs may become conflicted by the inability to proceed with what they believe are morally and ethically correct decisions due to external factors, such as inadequate compensation, resource limitations and difficult care situations [7, 81, 82, 85, 86].

To best understand and appreciate the effects on a HCP’s personhood, the lens of the RToP can further structure our findings.

#### The innate ring

As the Innate Ring contains belief systems informed by the HCP’s demographic features and spiritual and religious beliefs, exposure to practice across cultures and ethnic groups over a variety of settings can nurture cultural sensitivity and boost a more holistic perspective of patients [85] and their families, as well as their spiritual needs [80, 81, 85, 87] and concepts of dignity [82, 87]. Perceptions of death are also re-evaluated as a divine order facilitating the circle of life [7]. For example, nurses in Iran and Sweden reported that caring for dying patients led them to re-examine their lives and views on death whilst engaging in existential contemplation [7, 77].

#### The individual ring

Growing competence, experience and a developing sense of self help HCPs to boost adaptability [79, 80] and cope in the face of moral distress and growing costs of caring [82, 88]. Growth in the ability to cope with stressors is further reinforced when there are guided reflections [77, 81]. In Ercolani et al.’s [88] study of psychologists, physicians and nurses providing home palliative care, it was found that psychologists had improved coping by virtue of their formal training in recognising and regulating their emotional responses. In contrast, without effective support, the challenging aspects of HCPs’ experiences compound the effects of the costs of caring [86, 87].

#### The societal ring

Greater appreciation of a patient’s and their family’s sociocultural considerations fosters trust between HCPs and the patient and their family. This improves role clarity [79, 86] and collaboration that enhance patient outcomes [82, 89]. However, numerous factors exacerbate the effects of the costs of caring, including the exposure to health and economic disparities [85, 90]; professional, legal, sociocultural and systemic expectations and considerations [87, 88]; and poor interprofessional working practices and support [77, 89].

### Domain 3: challenges faced by HCPs

The challenges faced by HCPs can be broadly divided into subcategories, including personal, professional and systemic challenges.

#### Personal considerations

When the HCPs’ experiences, reflections and attempts at meaning-making align with their dominant beliefs, a feeling of *resonance* results; conversely, *dissonance* arises when there is conflict. Both states bear significant ramifications. *Dissonance* evokes a sense of helplessness, meaninglessness and fear of mortality that bleed into a HCP’s personal perspectives and relationships [88] and leave deeper issues unresolved [82]. *Dissonance* is further exacerbated when there is little support for patients and their families, a lack of guidance of HCP’s reflections and meaning-making [77] and/or conflicts between personal and professional beliefs [85, 88]. The challenges from *dissonance* can progress into feelings of burnout and exhaustion. Without proper channels to process unpleasant experiences, feelings of frustration can compound. Conversely, *resonance* is boosted when a holistic appreciation of patient needs and balanced roles and responsibilities foster greater insights, improved confidence [80], enhanced interprofessional team-working [82] and improved care delivery [79–81, 87]. These experiences were found to be shared across various healthcare professions providing home palliative care.

#### Professional considerations

HCPs across various professions face common professional challenges that test their expertise, resilience and adaptability [85, 88]. Balancing healthcare duties with complex and multifaceted patient needs [7, 81], coupled with inadequate support and training, limited resources and technical skills [30, 79, 81] and unrealistic patient and familial expectations [30], complicates coping [77, 84] and precipitates the costs of caring. In Johansen et al.’s [91] interprofessional study, it was found that both nurses and GPs shared similar concerns about facing out-of-office crises and a lack of communication between hospitals and primary care staff impeding smooth transitions of care.

Specific professions also face unique challenges. Nurses report feelings of isolation and a lack of support and guidance from physicians [82], often bearing full responsibility for managing not only medical needs, but also psychosocial and economic issues of their patients [7, 81]. CHWs who lack formal medical and nursing training also report specific limitations as they are often ill-equipped to handle emergencies, emotional distress and end-of-life needs [30]. This is in part due to the curative, rather than a palliative, intent in the type of care provided by CHWs [30].

Overall, HCPs grapple with inadequate training, limited resources, novice technical skills [30, 79, 81] and unrealistic expectations from patients and families—further compounding such difficulties [77, 84] and straining relationships between HCPs and families [30]. Volatile household environments, marked by conflict, substance dependency and antisocial behaviours, present additional sources of stress and obstacles to delivering effective care [84]. In contrast, an alignment of professional expectations and an acceptance of practice and professional limitations foster trust and strengthen care delivery [81, 87]​, enhance holistic care [79, 80] and reinforce professional confidence, competence [80] and multi-professional practice [82].

#### Systemic considerations

Poor care coordination between healthcare institutions and home settings presents a significant challenge in the homecare setting, resulting in fragmented care and limitations to care transitions [29, 85]. For instance, Johansen and Ervik’s [91] study revealed the challenges faced by GPs in fulfilling their roles as coordinators of care for palliative care patients previously managed by hospital specialists, particularly when inadequate information was documented. This was also recognised by nurses in the same study, who were concerned about compromises in the quality of medical care provided by GPs due to a lack of handover [91].

### Domain 4: support systems for HCPs

Personal and institutional support systems are essential for HCPs to navigate the personal, professional and institutional challenges of home-based palliative care. These support systems are found to be consistent across members of various health professions.

#### Personal coping strategies

Adaptive coping strategies, such as maintaining a positive attitude and problem-solving, reduce emotional exhaustion and distress [88] and the risks of the costs of caring [7, 81, 84], particularly when complemented with peer support [82] and effective work-life balance [77, 88].

#### Institutional support

A supportive team structure, monthly supervision sessions with an external expert and weekly multidisciplinary case discussions [92] promote effective care [77, 86], early identification of distress [86], self-awareness, self-care and professional satisfaction [30, 80]. Similarly, technology-enhanced communication with families, such as telemedicine and digital care plans, facilitates information exchange and timely support [87]. Additional training resources and educational opportunities are key to professional development [80], enhancing self-awareness, self-care and professional satisfaction [30, 80].

## Discussion

Our results reveal that providing home-based palliative care to adult oncology patients is fulfilling for HCPs, aligning with their vision of making “*a difference to somebody’s life*” [30, 79]. Many HCPs report a greater appreciation for life [7, 77], more holistic perspective of life and death and a heightened sense of personal accomplishment [83]. These experiences contribute to improved interprofessional working, deeper meaningful professional relationships with patients and their families and better understanding of the sociocultural considerations and needs of patients [30, 83]. In addition, HCPs report stronger ability in catering to the unique needs of patients [30, 83], navigating intense interpersonal dynamics, addressing crises, confronting end-of-life issues under challenging circumstances [21, 22, 28, 93, 94] and, in some cases, reducing hospital admissions [87]. Addressing our primary research question, ‘*What are the experiences of healthcare professionals caring for adult oncology patients receiving palliative care at home*?’, HCPs also reveal greater confidence [80] and cultural sensitivity [79–82, 85, 87].

In addressing our secondary research question ‘*How are healthcare professionals impacted by caring for adult oncology patients receiving palliative care at home?*’, HCPs also recognise the need for longitudinal support to contend with the costs of caring [77, 81, 82, 86–88]. These challenges are precipitated by health and economic disparities [85, 90]; professional, legal, sociocultural and systemic expectations [87, 88]; and poor working conditions and support [77, 89].

Therefore, beyond evidencing the costs of caring, this review underscores the need for greater purposeful, individualised, timely and longitudinal support for HCPs working in often difficult and solitary practices. This is critical to the shepherding of the HCP’s professional identity formation and thus their professional interactions and overall patient care. It is also prudent for tertiary institutions and specialist palliative care centers to frequently seek feedback from HCPs to gain insights and provide them with updated knowledge to improve expertise, such as the processes of hospice referral, end-of-life legislation and law (e.g. regarding advance directives) and symptom management [95, 96]. Further education and training can be provided at the undergraduate and post-graduate level through a continuing spiral curriculum with opportunities for experiential learning and longitudinal mentorship [22, 24, 93, 96–101], given that many HCPs still possess minimal palliative care training [95, 102, 103]. We draw on earlier work to suggest the that use of e-portfolios, in conjunction with a mentored training programme that supports immersive learning, is pivotal—particularly in the presence of changeable work settings where a structured practice environment may be challenging to create. Peer support and accessible and timely personalised support become all the more important, as are evaluations in changes to the HCP’s RToP. It is also our recommendation that future research focus on developing evidence-based support programmes, utilising frameworks such as the RToP, to ensure sustainable and fulfilling careers in this complex and evolving aspect of palliative care.

### Limitations

Majority of the literature focused on homecare nurses and, to a lesser extent, GPs. This may be due to the predominance of these professions in providing home palliative care compared to others, such as allied health professions. This limited scope may not fully capture the diverse experiences and contributions of the broader multidisciplinary team as palliative care continues to expand to include allied health professionals with formal palliative care training and roles [104, 105]. Whilst it was found that majority of the results were in concordance across various healthcare professions, we acknowledge that each healthcare profession has their unique role, practice and experiences. Thus, there may be reduced generalisability of the findings to other populations. Further, the inclusion of only English articles may have led to the omission of relevant non-English articles. Some findings, such as the ‘Doing Good Care’ theory, while constructed using proper methodology (such as interviews and thematic analyses) and enriching on their own, have not been further utilised and tested by subsequent studies. Thus, due to limited data, confirming the generalisability of this theory is challenging. However, by including such theories in our review, we aim to encourage further testing and validation. Lastly, despite efforts to reduce biases through the negotiated consensual approach and engagement of the expert team, individual bias may persist.

## Conclusion

This SEBA-guided scoping review has uncovered that HCPs working in palliative home settings garner positive motivations and experiences from their role, especially pertaining to healthy reflections on mortality and the belief in making a difference in others’ lives. However, they are also faced with emotional challenges at a personal and institutional level, where the effects of the costs of caring on personal and professional fronts are salient. Longitudinal and accessible personalised and organisational support, structured by frameworks such as the RToP, is thus necessary to sustain HCPs’ capacity to deliver compassionate and high-quality palliative care.

## Electronic supplementary material

Below is the link to the electronic supplementary material.

Supplementary Material 1: Additional File 1. PRISMA checklist

Supplementary Material 2: Additional File 2. Search strategy

Supplementary Material 3: Additional File 3. Tabulated summaries of included articles

## Data Availability

All data generated or analysed during this study are included in this published article and its supplementary information files.

## Abbreviations

CHW: Community Health Workers
GP: General Practitioner
HCP: Healthcare Professionals
PCC: Population, Concept, Context
RToP: Ring Theory of Personhood
SEBA: Systematic Evidence-Based Approach

## References

[1] Ho BJ, Akhileswaran R, Pang GSY, Koh GCH. An 11-year study of home hospice service trends in Singapore from 2000 to 2010. J Palliat Med. 2017;20(5):461–72. 28135117 10.1089/jpm.2016.0268

[2] Lo TJ, Neo PS, Peh TY, Akhileswaran R, Chen WT, Lee A, et al. Improving quality of palliative care through implementation of national guidelines for palliative care. J Palliat Med. 2019;22(11):1439–44. 30939062 10.1089/jpm.2018.0345

[3] Sijabat M, Dahlia D, Waluyo A. Experiences of palliative care nurses in providing home-based care for patient with advanced cancer. Enferm Clin. 2019;29(S2):413–7.

[4] Lim YX, Quah ELY, Chua KZY, Lin Ronggui CK, Govindasamy R, Ong SM, et al. A systematic scoping review on dignity assessment tools. J Pain Symptom Manage. 2024;67(4):e263–84. 38092260 10.1016/j.jpainsymman.2023.12.008

[5] Chua KZY, Quah ELY, Lim YX, Goh CK, Lim J, Wan DWJ, et al. A systematic scoping review on patients’ perceptions of dignity. BMC Palliat Care. 2022;21(1):118. 35787278 10.1186/s12904-022-01004-4 PMC9251939

[6] Quah ELY, Chua KZY, Lua JK, Wan DWJ, Chong CS, Lim YX, et al. A systematic review of stakeholder perspectives of dignity and assisted dying. J Pain Symptom Manage. 2023;65(2):e123–36. 36244639 10.1016/j.jpainsymman.2022.10.004

[7] Iranmanesh S, Axelsson K, Sävenstedt S, Häggström T. Caring for dying and meeting death: experiences of Iranian and Swedish nurses. Indian J Palliat Care. 2010;16(2):90–6. 21811355 10.4103/0973-1075.68405 PMC3144438

[8] Ministry of Health. Update on national strategy for palliative care [press release] 2024 [updated 19 March 2024. Available from: https://www.moh.gov.sg/newsroom/update-on-national-strategy-for-palliative-care]

[9] Li WW, Singh S, Keerthigha C. A cross-cultural study of filial piety and palliative care knowledge: moderating effect of culture and universality of filial piety. Front Psychol. 2021;12:787724. 34925189 10.3389/fpsyg.2021.787724 PMC8678124

[10] Sallnow ES. Collective social capital: a study of new public health and end-of-life care. 2018.

[11] Attridge C, Richardson H. 6 compassionate neighbours–an innovative model building caring communities. BMJ Supportive Palliat Care. 2018;8(3):362.

[12] Vijay D, Zaman S, Clark D. Translation of a community palliative care intervention: experience from West Bengal, India. Wellcome Open Res. 2018;3:66. 30116790 10.12688/wellcomeopenres.14599.1 PMC6069742

[13] Jain V. Palliative care in India: trials, tribulations, and future prospects. Mahatma Gandhi Inst Med Sci. 2018;23(2):55–8.

[14] Rajagopal M, Kumar S. A model for delivery of palliative care in India—the Calicut experiment. J Palliat Care. 1999;15(1):44–9. 10333664

[15] Sallnow L, Chenganakkattil S. The role of religious, social and political groups in palliative care in Northern Kerala. Indian J Palliat Care. 2005;11(1):10.

[16] Krishna LK. Decision-making at the end of life: a Singaporean perspective. Asian Bioeth Rev. 2011;3(2):118–26.

[17] Lalit Krishna M. The position of the family of palliative care patients within the decision-making process at the end of life in Singapore. Ethics Med. 2011;27(3):183.

[18] Krishna LKR, Alsuwaigh R, Miti PT, Wei SS, Ling KH, Manoharan D. The influence of the family in conceptions of personhood in the palliative care setting in Singapore and its influence upon decision making. Am J Hosp Palliat Care. 2014;31(6):645–54. 23946254 10.1177/1049909113500136

[19] Köbis N, Bonnefon J-F, Rahwan I. Bad machines corrupt good morals. Nat Hum Behav. 2021;5(6):679–85. 34083752 10.1038/s41562-021-01128-2

[20] Wong V, Krishna L. The meaning of food amongst terminally ill Chinese patients and families in Singapore. JMED Research. 2014;2014(2014):670628.

[21] Somasundaram N, Ibrahim H, Govindasamy R, Hamid NABA, Ong SYK, Krishna LKR. Caring for terminally ill patients: the impact on oncologists. BMC Palliat Care. 2024;23(1):231. 39342162 10.1186/s12904-024-01562-9 PMC11439311

[22] Ho CY, Lim N-A, Ong YT, Lee ASI, Chiam M, Gek GPL, et al. The impact of death and dying on the personhood of senior nurses at the National cancer centre Singapore (NCCS): a qualitative study. BMC Palliat Care. 2022;21(1):83. 35590293 10.1186/s12904-022-00974-9 PMC9121572

[23] Chan NPX, Chia JL, Ho CY, Ngiam LXL, Kuek JTY, Ahmad Kamal NHB, et al. Extending the ring theory of personhood to the care of dying patients in intensive care units. Asian Bioeth Rev. 2022;14(1):71–86. 34691261 10.1007/s41649-021-00192-0 PMC8526529

[24] Ong RSR, Wong RSM, Chee RCH, Quek CWN, Burla N, Loh CYL, et al. A systematic scoping review moral distress amongst medical students. BMC Med Educ. 2022;22(1):466. 35710490 10.1186/s12909-022-03515-3 PMC9203147

[25] Ibrahim H, Oyoun Alsoud L, West K, Maraka JO, Sorrell S, Harhara T, et al. Interventions to support medical trainee well-being after patient death: a scoping review. J Hosp Med. 2024;19(11):1044–52. 39154261 10.1002/jhm.13489

[26] Kuek JTY, Ngiam LXL, Kamal NHA, Chia JL, Chan NPX, Abdurrahman ABHM, et al. The impact of caring for dying patients in intensive care units on a physician’s personhood: a systematic scoping review. Philos Ethics Humanit Med. 2020;15(1):12. 33234133 10.1186/s13010-020-00096-1 PMC7685911

[27] Krishna LKR, Neo HY, Chia EWY, Tay KT, Chan N, Neo PSH, et al. The role of palliative medicine in ICU bed allocation in COVID-19: a joint position statement of the Singapore hospice council and the chapter of palliative medicine physicians. Asian Bioeth Rev. 2020;12(2):205–11. 32837552 10.1007/s41649-020-00128-0 PMC7262490

[28] Radha Krishna LK, Abdul Hamid NAB, Lim N-A, Ho CY, Ibrahim H. Journeying with the dying—lessons from palliative care physicians. Asian Bioeth Rev. 2024:1–23. 10.1007/s41649-024-00321-5 10.1007/s41649-024-00321-5 PMC12304369 40741311

[29] Atreya S, Patil C, Kumar R. Integrated primary palliative care model; facilitators and challenges of primary care/family physicians providing community-based palliative care. J Family Med Prim Care. 2019;8(9):2877–81. 31681659 10.4103/jfmpc.jfmpc_653_19 PMC6820380

[30] Potts M, Cartmell K, Nemeth L, Qanungo S. A qualitative evaluation of a home-based palliative care program utilizing community health workers in India. Indian J Palliat Care. 2019;25(2):181–9. 31114101 10.4103/IJPC.IJPC_166_18 PMC6504743

[31] Sinnathamby A, Ong YT, Lim SX, Hiew AWH, Ng SY, Chee JH, et al. Concepts of suffering at the end of life amongst emergency, palliative care and geriatric medicine physicians in Malaysia [online ahead of print]. Am J Hosp Palliat Care. 2025:10499091251317725. 10.1177/10499091251317725 10.1177/10499091251317725 PMC12627250 39879618

[32] Sinnathamby A, Ibrahim H, Ong YT, Ravindran N, Wan DWJ, Tan JH, et al. Towards a theory of compassion fatigue in palliative care and oncology: a systematic scoping review. Am J Hosp Palliat Care. 2025:10499091251315183. 10.1177/10499091251315183 10.1177/10499091251315183 PMC12705876 39825792

[33] Ho CY, Lim N-A, Rahman NDA, Chiam M, Zhou JX, Phua GLG, et al. Physician-patient boundaries in palliative care. BMC Palliat Care. 2023;22(1):41. 37055737 10.1186/s12904-023-01161-0 PMC10099695

[34] Ong YT, Sinnathamby A, Tan JH, Ravindran N, Lim SX, Hiew AWH, et al. Towards a clinically relevant appreciation of the cost of caring: a study of palliative care physicians in Malaysia. Am J Hosp Palliat Care. 2024;7:10499091241298281. 10.1177/10499091241298281 39508141

[35] Joshi I, Zemel R. COVID-19 and the new hidden curriculum of moral injury and compassion fatigue. Am J Hosp Palliat Care. 2024;42(2):133–9. 38768440 10.1177/10499091241253283

[36] Bird A, Tomescu O, Oyola S, Houpy J, Anderson I, Pincavage A. A curriculum to teach resilience skills to medical students during clinical training. MedEdPORTAL. 2020;16:10975. 33015355 10.15766/mep_2374-8265.10975 PMC7526502

[37] Dias AR, Fernandes SM, Fialho-Silva I, Cerqueira-Silva T, Miranda-Scippa Â, Almeida AG. Burnout syndrome and resilience in medical students from a Brazilian public college in Salvador, Brazil. Trends Psychiatry Psychother. 2022;44:e20200187. 34139116 10.47626/2237-6089-2020-0187 PMC9907392

[38] Gheihman G, Cooper C, Simpkin A. Everyday resilience: practical tools to promote resilience among medical students. J Gen Intern Med. 2019;34(4):498–501. 30535748 10.1007/s11606-018-4728-8 PMC6445902

[39] Houpy JC, Lee WW, Woodruff JN, Pincavage AT. Medical student resilience and stressful clinical events during clinical training. Med Educ Onlin. 2017;22(1):1320187. 10.1080/10872981.2017.1320187 PMC5419301 28460570

[40] Liu Y, Cao Z. The impact of social support and stress on academic burnout among medical students in online learning: the mediating role of resilience. Front Public Health. 2022;10:938132. 35937240 10.3389/fpubh.2022.938132 PMC9355500

[41] Mugford H, O’connor C, Danelson K, Popoli D. Medical students’ perceptions and retention of skills from active resilience training. Fam Med. 2022;54(3):213–5. 35303303 10.22454/FamMed.2022.462706

[42] Duarte I, Alves A, Coelho A, Ferreira A, Cabral B, Silva B, et al. The mediating role of resilience and life satisfaction in the relationship between stress and burnout in medical students during the COVID-19 pandemic. Int J Environ Res Public Health. 2022;19(5):2822. 35270518 10.3390/ijerph19052822 PMC8910345

[43] Forycka J, Pawłowicz-Szlarska E, Burczyńska A, Cegielska N, Harendarz K, Nowicki M. Polish medical students facing the pandemic-assessment of resilience, well-being and burnout in the COVID-19 era. PLoS ONE. 2022;17(1):e0261652. 35073318 10.1371/journal.pone.0261652 PMC8786167

[44] Rahimi B, Baetz M, Bowen R, Balbuena L. Resilience, stress, and coping among Canadian medical students. Can Med Educ J. 2014;5(1):e5–12. 26451221 PMC4563614

[45] Zila-Velasque JP, Grados-Espinoza P, Chuquineyra BS, Diaz-Vargas M, Sierra Calderón GS, Choquegonza S, et al. Resilience, sleep quality and sleepiness in Peruvian medical students: a multicenter study. Front Psychiatry. 2024;15:1284716. 39211539 10.3389/fpsyt.2024.1284716 PMC11358107

[46] Jordan RK, Shah SS, Desai H, Tripi J, Mitchell A, Worth RG. Variation of stress levels, burnout, and resilience throughout the academic year in first-year medical students. PLoS ONE. 2020;15(10):e0240667. 33057410 10.1371/journal.pone.0240667 PMC7561113

[47] Danielsen BV, Sand AM, Rosland JH, Førland O. Experiences and challenges of home care nurses and general practitioners in home-based palliative care–a qualitative study. BMC Palliat Care. 2018;17:1–13. 10.1186/s12904-018-0350-0 PMC6052702 30021583

[48] Quigley DD, McCleskey SG. Improving care experiences for patients and caregivers at end of life: a systematic review. Am J Hosp Palliat Care. 2021;38(1):84–93. 32551966 10.1177/1049909120931468 PMC8526304

[49] Hebert RS, Schulz R, Copeland VC, Arnold RM. Preparing family caregivers for death and bereavement. Insights from caregivers of terminally ill patients. J Pain Symptom Manage. 2009;37(1):3–12. 18538977 10.1016/j.jpainsymman.2007.12.010

[50] Sarmento VP, Gysels M, Higginson IJ, Gomes B. Home palliative care works: but how? A meta-ethnography of the experiences of patients and family caregivers. BMJ Support Palliat Care. 2017;7(4):390–403. 10.1136/bmjspcare-2016-001141 28232515

[51] Danielsen BV, Sand AM, Rosland JH, Førland O. Experiences and challenges of home care nurses and general practitioners in home-based palliative care–a qualitative study. BMC Palliat Care. 2018;17(1):95. 30021583 10.1186/s12904-018-0350-0 PMC6052702

[52] Weiss CR, Florell M, Oman K, Sousa K. Concept analysis of disenfranchised grief within a nursing paradigm: to awaken our caring humanity. Int J Hum Caring. 2023;27(2):92–103.

[53] Daniels CL. Social workers as key informants: Identifying disenfranchised grief in nursing home interdisciplinary teams during COVID-19 [dissertation]. Miami Shores, Florida: Barry University; 2023.

[54] Wandke S, Fuehres H, Rutenkroeger M, Lang K, Haerter M, Oechsle K et al. Professional grief in cancer care-a scoping review [preprint]. medRxiv. 2024:24311612. [Available from: https://www.medrxiv.org/content/24311610.24311101/24312024.24311608.24311607.24311612v24311611]. 10.1002/pon.70156 PMC12031695 40280892

[55] Hawes FM, Wang S. A comparative study of organizational grief support and burnout among nursing home staff. Gerontologist. 2024;64(8):gnae065. 38832394 10.1093/geront/gnae065

[56] Prompantakorn P, Angkurawaranon C, Pinyopornpanish K, Chutarattanakul L, Aramrat C, Pateekhum C, et al. Palliative performance scale and survival in patients with cancer and non-cancer diagnoses needing a palliative care consultation: a retrospective cohort study. BMC Palliat Care. 2021;20(1):74. 34039322 10.1186/s12904-021-00773-8 PMC8157447

[57] Ng YX, Koh ZYK, Yap HW, Tay KT, Tan XH, Ong YT, et al. Assessing mentoring: a scoping review of mentoring assessment tools in internal medicine between 1990 and 2019. PLoS ONE. 2020;15(5):e0232511. 32384090 10.1371/journal.pone.0232511 PMC7209188

[58] Bousquet J, Schunemann HJ, Samolinski B, Demoly P, Baena-Cagnani CE, Bachert C, et al. Allergic rhinitis and its impact on asthma (ARIA): achievements in 10 years and future needs. J Allergy Clin Immunol. 2012;130(5):1049–62. 23040884 10.1016/j.jaci.2012.07.053

[59] Bok C, Ng CH, Koh JWH, Ong ZH, Ghazali HZB, Tan LHE, et al. Interprofessional communication (IPC) for medical students: a scoping review. BMC Med Educ. 2020;20(1):372. 33081781 10.1186/s12909-020-02296-x PMC7574565

[60] Ahmad Kamal NH, Tan LHE, Wong RSM, Ong RRS, Seow REW, Loh EKY, et al. Enhancing education in palliative medicine: the role of systematic scoping reviews. Palliat Med Care. 2020;7(1):1–11.

[61] Ong RRS, Seow REW, Wong RSM, Loh EKY, Ahmad Kamal NH, Mah ZH, et al. A systematic scoping review of narrative reviews in palliative medicine education. Palliat Med Care. 2020;7(1):1–22.

[62] Mah ZH, Wong RSM, Seow REW, Loh EKY, Ahmad Kamal NH, Ong RRS, et al. A systematic scoping review of systematic reviews in palliative medicine education. Palliat Med Care. 2020;7(1):1–12.

[63] Wyatt TR, Rockich-Winston N, White D, Taylor TR. Changing the narrative: a study on professional identity formation among Black/African American physicians in the US. Adv Health Sci Educ Theory Pract. 2021;26(1):183–98. 32572728 10.1007/s10459-020-09978-7

[64] Pring R. The ‘false dualism’ of educational research. J Philos Educ. 2000;34(2):247–60.

[65] Crotty M. The foundations of social research: meaning and perspective in the research process. London, UK: SAGE; 1998. p. 256.

[66] Ford DW, Downey L, Engelberg R, Back AL, Curtis JR. Discussing religion and spirituality is an advanced communication skill: an exploratory structural equation model of physician trainee self-ratings. J Palliat Med. 2012;15(1):63–70. 22242716 10.1089/jpm.2011.0168

[67] Schick-Makaroff K, MacDonald M, Plummer M, Burgess J, Neander W. What synthesis methodology should I use? A review and analysis of approaches to research synthesis. AIMS Public Health. 2016;3(1):172–215. 29546155 10.3934/publichealth.2016.1.172 PMC5690272

[68] Deshpande AD, Sanders Thompson VL, Vaughn KP, Kreuter MW. The use of sociocultural constructs in cancer screening research among African Americans. Cancer Control. 2009;16(3):256–65. 19556966 10.1177/107327480901600308 PMC3614350

[69] Stephens MB, Bader KS, Myers KR, Walker MS, Varpio L. Examining professional identity formation through the ancient art of mask-making. J Gen Intern Med. 2019;34(7):1113–5. 30891691 10.1007/s11606-019-04954-3 PMC6614309

[70] Burla N, Ong RSR, Chee RCH, Wong RSM, Neo SY, Abdul Hamid NAB, et al. A systematic scoping review on group non-written reflections in medical education. BMC Med Educ. 2024;24(1):1119. 39390436 10.1186/s12909-024-06117-3 PMC11468106

[71] Phua GLG, Owyong JLJ, Leong ITY, Goh S, Somasundaram N, Poon EYL, et al. A systematic scoping review of group reflection in medical education. BMC Med Educ. 2024;24(1):398. 38600515 10.1186/s12909-024-05203-w PMC11007913

[72] Lim JY, Ong SYK, Ng CYH, Chan KLE, Wu SYEA, So WZ, et al. A systematic scoping review of reflective writing in medical education. BMC Med Educ. 2023;23(1):12. 36624494 10.1186/s12909-022-03924-4 PMC9830881

[73] Quek CWN, Ong RRS, Wong RSM, Chan SWK, Chok AK, Shen GS, et al. Systematic scoping review on moral distress among physicians. BMJ Open. 2022;12(9):e064029. 36691160 10.1136/bmjopen-2022-064029 PMC9442489

[74] Sandelowski M, Barroso J. Handbook for synthesizing qualitative research. New York, NY: Springer Publishing Company; 2006. p. 312.

[75] Clarke V, Braun V. Thematic analysis. J Posit Psychol. 2017;12(3):297–8.

[76] Hsieh H-F, Shannon SE. Three approaches to qualitative content analysis. Qual Health Res. 2005;15(9):1277–88. 16204405 10.1177/1049732305276687

[77] Kaup J, Höög L, Carlsson ME. Care for dying patients at midlife. J Hosp Palliat Nurs. 2016;18(6):564–71.

[78] Moss PA, Haertel EH. Engaging methodological pluralism. In: Gitomer D, Bell C, editors. Handbook of research on teaching. 5 ed. Washington, DC: American Educational Research Association; 2016. pp. 127–247.

[79] Griffiths J, Ewing G, Rogers M, Barclay S, Martin A, McCabe J, et al. Supporting cancer patients with palliative care needs: district nurses’ role perceptions: district nurses’ role perceptions. Cancer Nurs. 2007;30(2):156–62. 17413782 10.1097/01.NCC.0000265013.63547.4a

[80] Johnston G, Davison D, Reilly P. Educational needs in palliative care: a survey of gps and community nurses. Eur J Gen Pract. 2001;7(3):99–103.

[81] Bertero C. District nursers’ perceptions of palliative care in the home. Am J Hosp Palliat Care. 2002;19(6):387–91. 12442973 10.1177/104990910201900608

[82] Sandgren A, Thulesius H, Petersson K, Fridlund B. Doing good care - a study of palliative home nursing care. Int J Qual Stud Health Well-being. 2007;2(4):227–35.

[83] Ndiok A, Ncama B. A qualitative study of home visiting as a palliative care strategy to follow-up cancer patients by nurses in clinical setting in a developing country. Scand J Caring Sci. 2019;33(1):185–96. 30295326 10.1111/scs.12619

[84] Wilson C, Griffiths J, Ewing G, Connolly M, Grande G. A qualitative exploration of district nurses’ care of patients with advanced cancer. Cancer Nurs. 2014;37(4):310–5. 23945144 10.1097/NCC.0b013e31829a9a56

[85] Hassankhani H, Rahmani A, Best A, Taleghani F, Sanaat Z, Dehghannezhad J. Barriers to home-based palliative care in people with cancer: a qualitative study of the perspective of caregivers. Nurs Open. 2020;7(4):1260–8. 32587746 10.1002/nop2.503 PMC7308678

[86] Varani S, Ostan R, Franchini L, Ercolani G, Pannuti R, Biasco G, et al. Caring for advanced cancer patients at home during COVID-19 outbreak: burnout and psychological morbidity among palliative care professionals in Italy. J Pain Symptom Manage. 2021;61(2):e4–12. 33249082 10.1016/j.jpainsymman.2020.11.026 PMC7691143

[87] Spelten E, Timmis J, Heald S, Duijts SFA. Rural palliative care to support dying at home can be realised; experiences of family members and nurses with a new model of care. Aust J Rural Health. 2019;27(4):336–43. 31429138 10.1111/ajr.12518

[88] Ercolani G, Varani S, Peghetti B, Franchini L, Malerba MB, Messana R, et al. Burnout in home palliative care: what is the role of coping strategies? J Palliat Care. 2020;35(1):46–52. 30727827 10.1177/0825859719827591

[89] Brogaard T, Jensen AB, Sokolowski I, Olesen F, Neergaard MA. Who is the key worker in palliative home care? Scand J Prim Health Care. 2011;29(3):150–6. 21861601 10.3109/02813432.2011.603282 PMC3347960

[90] Beccaro M, Lora Aprile P, Scaccabarozzi G, Cancian M, Costantini M. Survey of Italian general practitioners: knowledge, opinions, and activities of palliative care. J Pain Symptom Manage. 2013;46(3):335–44. 23195391 10.1016/j.jpainsymman.2012.08.020

[91] Johansen M-L, Ervik B. Talking together in rural palliative care: a qualitative study of interprofessional collaboration in Norway. BMC Health Serv Res. 2022;22(1):314. 35255918 10.1186/s12913-022-07713-z PMC8900365

[92] Asaumi K, Oki M, Murakami Y. Timely identification of patients with cancer and family caregivers in need of end-of-life discussions by home-visit nurses in Japan: a qualitative descriptive study. Glob Qual Nurs Res. 2023;10:1–10. 10.1177/23333936221146048 PMC9834930 36644373

[93] Tay J, Compton S, Phua G, Zhuang Q, Neo S, Lee G, et al. Perceptions of healthcare professionals towards palliative care in internal medicine wards: a cross-sectional survey. BMC Palliat Care. 2021;20(1):101. 34193142 10.1186/s12904-021-00787-2 PMC8247075

[94] Singapore Hospice C. 2020 SHC key survey findings of awareness of hospice & palliative care among healthcare professionals in Singapore. 2020 [Available from: https://www.singaporehospice.org.sg/e-library/docs/2020-shc-survey-key-findings-of-awareness-of-hospice-palliative-care-among-healthcare-professionals-in-singapore-2/

[95] Niaré D, Robert G, Rocquevieille A, De Geyer L, Frin M, Pennec S, et al. General practitioners and palliative care practices: a better knowledge of specific services is still needed. BMC Health Serv Res. 2024;24(1):832. 39044274 10.1186/s12913-024-11266-8 PMC11264423

[96] O’Brien H, Kruger C, Ravindrarasan S, Kiely F, Foley T. Perceived palliative care education needs of GP trainees: a national study. J Pain Symptom Manage. 2023;66(4):320–e327313. 37380146 10.1016/j.jpainsymman.2023.06.021

[97] Krishna LKR, Tan LHE, Ong YT, Tay KT, Hee JM, Chiam M, et al. Enhancing mentoring in palliative care: an evidence based mentoring framework. J Med Educ Curric Dev. 2020;7:2382120520957649. 33015366 10.1177/2382120520957649 PMC7517982

[98] Krishna LKR. Towards an evidence based approach to novice mentoring in academic clinical practice: lessons from internal medicine and practical applications for palliative medicine [dissertation]. Liverpool, United Kingdom: The University of Liverpool. 2020.

[99] Koh EYH, Koh KK, Renganathan Y, Krishna L. Role modelling in professional identity formation: a systematic scoping review. BMC Med Educ. 2023;23(1):194. 36991373 10.1186/s12909-023-04144-0 PMC10052869

[100] Goh S, Wong RSM, Quah ELY, Chua KZY, Lim WQ, Ng ADR, et al. Mentoring in palliative medicine in the time of covid-19: a systematic scoping review. BMC Med Educ. 2022;22(1):359. 35545787 10.1186/s12909-022-03409-4 PMC9094135

[101] Chia EWY, Tay KT, Xiao S, Teo YH, Ong YT, Chiam M, et al. The pivotal role of host organizations in enhancing mentoring in internal medicine: a scoping review. J Med Educ Curric Dev. 2020;7:2382120520956647. 33062895 10.1177/2382120520956647 PMC7536487

[102] Callinan J. Hospice educators’ perspectives on e-learning in palliative care education in Ireland. Int J Palliat Nurs. 2024;30(10):536–46. 39422926 10.12968/ijpn.2024.30.10.536

[103] World Health Organization. Strengthening of palliative care as a component of integrated treatment throughout the life course. J Pain Palliat Care Pharmacother. 2014;28(2):130–4. 24779434 10.3109/15360288.2014.911801

[104] Morgan D, Rawlings D, Button E, Tieman J. Allied health clinicians’ understanding of palliative care as it relates to patients, caregivers, and health clinicians: a cross-sectional survey. J Allied Health. 2019;48(2):127–33. 31167015

[105] Tieman J, Morgan D, Jones K, Gordon S, Chakraborty A. Allied health professionals’ contribution to care at end of life in aged care settings. Aust J Prim Health. 2023;29(4):341–8. 36740449 10.1071/PY22178

