# Supplementary material for: Experiences of healthcare professionals providing palliative care in home settings - a scoping review
Source: BMC Palliat Care. 2025 Mar 28;24:83. doi: 10.1186/s12904-025-01728-z (PMC11951797; doi:10.1186/s12904-025-01728-z)
Supplement: Supplementary file 2 — Supplementary Material 2: Additional File 2. Search strategy [file 12904_2025_1728_MOESM2_ESM.docx]

**Embase**

| Caregivers | ('caregiver'/mj OR 'caregiver burden'/mj OR care*:ti,ab OR caring:ti,ab) AND |
| --- | --- |
| Cancer Patients | ('neoplasm'/mj OR neoplas*:ti,ab OR oncolog*:ti,ab OR cancer*:ti,ab OR carcinoma*:ti,ab OR tumor*:ti,ab OR tumour*:ti,ab) AND |
| Palliative care/terminally ill | ('palliative therapy'/mj OR 'terminal care'/mj OR 'end of life':ti,ab OR end-of-life:ti,ab OR palliat*:ti,ab OR ‘terminal care’:ti,ab OR terminal:ti,ab)  AND |
| Home | (‘home care'/mj OR ‘home’/mj OR home*:ti,ab OR house*:ti,ab) |
|  | 6414 results (2000-2024) searched 02/10/2024 |

**PubMed**

| Caregivers | ("caregivers"[MeSH] OR "caregiver burden"[MeSH] OR care*[tiab] OR caring[tiab]) AND |
| --- | --- |
| Cancer Patients | (“Neoplasms”[Mesh] OR neoplas*[tiab] OR oncolog*[tiab] OR cancer*[tiab] OR carcinoma*[tiab] OR tumor*[tiab] OR tumour*[tiab]) AND |
| Palliative care/terminally ill | ("Palliative care"[MeSH] OR "Palliative Medicine"[MeSH] OR "Terminal Care"[MeSH] OR "end of life"[tiab] OR “end-of-life”[tiab] OR palliat*[tiab] OR "terminal care"[tiab] OR terminal[tiab] OR late[tiab] OR final[tiab]) AND |
| Home | ("home care services"[MeSH] OR "home nursing"[MeSH] OR “home environment”[Mesh] OR “transitional care”[Mesh] OR home*[tiab] OR house*[tiab]) |
|  | 4170 results (2000-2024) searched 01/10/2024 |

**CINAHL**

| Caregivers | (MM("caregivers” OR "caregiver burden") OR TI (care* OR caring) OR AB (care* OR caring)) AND |
| --- | --- |
| Cancer Patients | (MM(“Neoplasms”) OR TI (neoplas* OR oncolog* OR cancer* OR carcinoma* OR tumor* OR tumour*) OR AB (neoplas* OR oncolog* OR cancer* OR carcinoma* OR tumor* OR tumour*)) AND |
| Palliative care/terminally ill | (MM("Palliative care” OR "Palliative Medicine" OR "Terminal Care") OR TI("end of life" OR “end-of-life” OR palliat* OR "terminal care" OR terminal OR late OR final) OR AB("end of life" OR “end-of-life” OR palliat* OR "terminal care" OR terminal OR late OR final)) AND |
| Home | (MM("home health care" OR "home nursing" OR “home environment” OR “transitional care”) OR TI(home* OR house*) OR AB(home* OR house*)) |
|  | 2237 (2000-2024) searched 01/10/2024 |

**PsycINFO**

| Caregivers | (exp Caregivers/ or exp caregiver burden/ or (care* or caring).ti,ab.) AND |
| --- | --- |
| Cancer Patients | (exp Terminal Cancer/ or exp Oncology/ or exp Cancers/ or exp Carcinomas/ or exp Tumors/ or (Neoplasm* or Tumour*).ti,ab.) AND |
| Palliative care/terminally ill | (exp Palliative Care/ or exp "End of Life Care"/ or ("end of life" or end-of-life or palliat* or terminal care or terminal or late or final).ti,ab.) AND |
| Home | (exp Home Care/ or exp Home Environment/ or (Home* or House*).ti,ab.) |
|  | 671 results, 2000-2024, 01/10/2024 |

**Scopus**

| Caregivers | (care* OR caring) W/7 |
| --- | --- |
| Cancer Patients | (neoplas* OR oncolog* OR cancer* OR carcinoma* OR tumor* OR tumour*) W/7 |
| Palliative care/terminally ill | ("end of life" OR “end-of-life" OR palliat* OR terminal) W/7 |
| Home | (home* OR house*) |
|  | 979 (2000-2024) searched 01/10/2024 |
