# Supplementary material for: Experiences of healthcare professionals providing palliative care in home settings - a scoping review
Source: BMC Palliat Care. 2025 Mar 28;24:83. doi: 10.1186/s12904-025-01728-z (PMC11951797; doi:10.1186/s12904-025-01728-z)
Supplement: Supplementary file 3 — Supplementary Material 3: Additional File 3. Tabulated summaries of included articles [file 12904_2025_1728_MOESM3_ESM.docx]

**Additional File 3. Tabulated Summaries of Included Articles**

| **No.** | **Title** | **Year** | **Authors** | **Introduction** | **Methods** | **Results** | **Conclusions** | **Population** | **Study Type** | **MERSQI**  **/COREQ** |
| --- | --- | --- | --- | --- | --- | --- | --- | --- | --- | --- |
| **1** | Educational needs in palliative care: A survey of GPs and community nurses | 2001 | Johnston, G.; Davison, D.; Reilly, P. | The aim of this study was to carry out an educational needs assessment in palliative care of general practitioners and community nurses in Northern Ireland. | Semi-structured postal questionnaire was sent to 1018 GPs and 750 community nurses with a reminder approximately one month later. y. Topics covered included staff's clinical and operational policy in palliative care, their use of specialist palliative care services, their attitude to guidelines and standards, their perception of their own competency in palliative care skills, their perceived need for further training and their preference for how this should be addressed. The questionnaire included a compilation of specially developed questions as well as some used in previous studies of educational needs in primary care. | 611 GPs and 497 nurses replied giving a response rate of 60% for the doctors and 66% for nurses. 52% of doctors and 94% of nurses expressed a special interest in palliative care. Though the majority of both groups felt confident in their palliative care skills, 83% of doctors and 95% of nurses said they would be likely to undertake further training in palliative care. Preferences for the topics and type of further training were similar for the two groups. The most popular topics for further training for both groups were pain and symptom control, bereavement care and research and audit methods. Preferred methods of further training included specialist lectures, discussion with specialists, hands-on experience in a hospice and multidisciplinary team meetings. Perceived barriers to further training were lack of time, lack of locally available courses, expense of providing locums or of self-funding courses and lack of recognition from management. | Primary care staff have expressed a keenness to provide community palliative care and provide it well. | GP and community nurses | Questionnaire study | 8 |
| **2** | District nursers' perceptions of palliative care in the home | 2002 | Bertero, Carina | The purpose of this paper is to explore the meaning of palliative care according to the experience of district nurses in Sweden. | Six district nurses were interviewed, and the transcripts were analyzed using Giorgi’s phenomenology. The guiding  questions for the study, which each nurse was asked, were  • What does it mean for you to care for and about cancer patients?  • Are there any difficulties when caring for cancer patients in their homes?  • Are there any advantages when caring for cancer patients in their homes? | Four major themes of the experience with palliative care in the home were identified in this study: challenge, control, frustration, and relationships, all of which were factors affecting commitment to the job. | This study points to exactly this; the district nurses must be offered resources and education to be able to fulfill their commitment to supplying good palliative care in the home. In order to care effectively for seriously ill and dying people, district nurses themselves need support. | District nurse | Interview study | 13 |
| **3** | "Doing good care" - A study of palliative home nursing care | 2007 | Sandgren, A.; Thulesius, H.; Petersson, K.; Fridlund, B. | The aim of this study was to develop a classic grounded theory of palliative home nursing care and we analysed interviews and data related to the behavior of community nurses caring for palliative cancer patients. | We interviewed 32 female nurses and one male nurse, and 30 of them had more than 10 years of working experience at hospitals or/and in homecare. The interviews began with ‘‘Tell me what it’s like to care for palliative cancer patients’’. Ideas emerged of what to ask next while interviewing, and more specific questions for later interviews while analysing such as ‘‘Tell me about a difficult caring situation’’, ‘‘How do you handle difficult situations?’’ and ‘‘Tell me about a caring situation that went well’’. This procedure is a property of theoretical sampling. ‘‘Theoretical sampling is the process of data collection for generating theory whereby the analyst jointly collects, codes, and analyses his data and decides what data to collect next and where to find them, in order to develop his theory as it emerges’’ (Glaser, 1978, p. 36). The first ten interviews were taperecorded and transcribed, but for the later interviews, only field notes were taken according to classic grounded theory (Glaser, 1998, 2001). Interviews lasted between 45 and 90 min. By the end of the study, interviews were shorter owing to the delimiting properties of grounded theory | In this grounded theory, we found that the main concern for nurses caring for palliative cancer patients in basic homecare is their desire of doing good. The nurses use different caring behaviors to handle this ‘‘do-good-desire’’ with anticipatory caring as the optimal behavior. However, the most common caring behavior is Momentary caring. Stagnated care is less common but can cause serious consequences for the nurses, patients, and relatives. When nurses fail in doing good, depending on their caring behavior, they use different strategies such as Emotional Shielding, Emotional Processing and Emotional Postponing | All nurses should have the opportunity to give Anticipatory care, which is the optimal way of caring. Health care providers thus need to increase palliative homecare resources as well as the status of caregivers through adequate recognition. The first step could be helping nurses to avoid Stagnated caring by giving them recognition. Then with the right resources, both internal and external, anticipatory caring is possible. | Community nurse | Interview study | 11 |
| **4** | Supporting cancer patients with palliative care needs: district nurses' role perceptions: district nurses' role perceptions | 2007 | Griffiths, J.; Ewing, G.; Rogers, M.; Barclay, S.; Martin, A.; McCabe, J.; Todd, C. | The aim of this study was to examine UK district nurses’ perceptions of their role in supporting palliative care cancer patients. | Semistructured interviews were conducted with 34 district nurses. Data were analyzed thematically, with assistance from Atlas/ti. The focus of this article is the qualitative interviews with  district nurses who completed symptom assessments for cancer  patients | A dominant theme emerging from the interviews was ambiguity in the district nurses’ supportive role in early palliative care. District nurses discussed the importance of making contact early on to support cancer patients and their families but had difficulty articulating this ‘‘support.’’ Ambiguity, lack of confidence, and perceived skill deficits presented district nurses with dilemmas that were difficult to resolve. | District nurses have great potential for meeting cancer patients’ supportive and palliative care needs, a potential not currently realized. Education alone is unlikely to improve practice without an understanding of the tensions faced by district nurses in their work. Recognizing and addressing dilemmas in the everyday work of district nurses is central to moving practice forward. | District nurse | Interview study | 14 |
| **5** | Caring for dying and meeting death: experiences of Iranian and Swedish nurses | 2010 | Iranmanesh, S.; Axelsson, K.; S√§venstedt, S.; H√§ggstr√∂m, T. | Our world is rapidly becoming a global community, which creates a need to further understand the universal phenomena of death and professional caring for dying persons. This study thus was conducted to describe the meaning of nurses’ experiences of caring for dying people in the cultural contexts of Iran and Sweden. | Using a phenomenological approach, phenomenon of caring for dying people was studied. Eight registered nurses who were working in oncology units in Tehran, Iran and eight registered nurses working in hospital and home care in North part of Sweden were interviewed. The interviews were analyzed using the principles of phenomenological hermeneutics. The participants were asked to narrate their experience of caring for dying people. Clarifying and encouraging questions were used, such as “Please, explain more about…”? or “Can you give an example”? The interviews were conducted in the informants’ mother tongue and by the members of target language (S.I in Iran and T. H., and S. S in Sweden). During the interviews, the researchers tried to strike a balance between listening to the stories told by the participants and keeping the focus of the stories on the aim. The interviews lasted between 45 and 60 min. | The findings were formulated based on two themes included: (1) “Sharing space and time to be lost”,  and (2) “Caring is a learning process. | The results showed that being with dying people raise an ethical demand that calls for personal and professional response, regardless of sex, culture or context. The physical and organizational context must be supportive and enable nurses to stand up to the demands of close relationships. Specific units and teamwork across various personnel seem to be a solution that is missing in Iran. | Nurse | Interview study | 9 |
| **6** | Survey of Italian general practitioners: Knowledge, opinions, and activities of palliative care | 2013 | Beccaro, M.; Aprile, P. L.; Scaccabarozzi, G.; Cancian, M.; Costantini, M. | This survey aimed to investigate the knowledge, opinions, and activities of Italian GPs regarding palliative care | A telephone survey of 1690 GPs was performed. Information was gathered through an ad hoc questionnaire. The questionnaire covered three areas and included six questions on knowledge, seven questions on opinions, and three questions on the activities of Italian GPs regarding palliative care. All questions were multiple choice (more than one answer could be chosen when relevant) and, in some cases, measured using a 10-point Likert scale. The questionnaire also included questions about the personal and professional characteristics of the GPs included in the survey (gender, age, geographic area, years of experience, and number of end-of-life patients per year). | Regarding knowledge, 25% of GPs recognized a correct definition of palliative care, 41% the objectives of palliative care, 66% that palliative care should be provided by  a multiprofessional team including GPs, and 60% that in-home care for patients at  the end of life requires an individual plan care. Furthermore, 92% of them reported  that ‘‘there is no maximum daily morphine dose for the management of pain.’’  Regarding opinions, most of the GPs strongly agreed that for patients at the end of  life, the GPs’ duties included availability during working hours to break bad news to  patients and families and to collaborate with the multiprofessional team in  establishing an individual care plan. Finally, regarding activities, most GPs reported  that, in their daily practice with patients at the end of life, they discontinue the drugs  that are not beneficial to symptom management and seek advice from palliative care  physicians when symptom management is ineffective. | This survey reveals the uncertainty of GPs regarding many theoretical issues but a strong willingness to integrate with the multiprofessional palliative care team. To further enhance the skills of GPs and facilitate the collaboration with palliative care services, it might be useful to realize ad hoc training schemes tailored to the different organizational procedures of in-home palliative care services. | GP | Questionnaire study | 8 |
| **7** | A Qualitative Exploration of District Nurses' Care of Patients With Advanced Cancer | 2014 | Wilson, Charlotte; Griffiths, Jane; Ewing, Gail; Connolly, Michael; Grande, Gunn | The objective of this study was to explore DNs’ experiences of supporting patients within families | Focus groups were conducted with 40 DNs from 4 areas in the United Kingdom. The groups were digitally recorded and facilitated by researchers using a flexible topic guide. The facilitator asked contributors to expand on areas while avoiding asking leading questions. Participants were asked: ‘‘How do you provide support to patients with cancer?’’ ‘‘What are the challenges or difficulties associated with this work?’’ As the focus groups progressed, key themes emerged and were introduced into the topic guide | Case-load complexity (household volatility) and family dynamics posed distinct challenges for nurses supporting patients. Many family members struggled with accepting the patients’ prognosis and were complicit in withholding information. At times, this foreclosed a consideration of palliative options | Carers provide a great deal of positive supportive care within the home. However, for some, the home is characterized by conflict rather than consensus. Complexities surrounding family relationships pose a distinctive and challenging environment for DNs | District nurse | Focus Group Study | 16 |
| **8** | Care for Dying Patients at Midlife | 2016 | Kaup, Jaana; H√∂√∂g, Lina; Carlsson, Maria E. | The aim of the study was to highlight nurses’ experiences of caring for patients in palliative care at midlife, and describe their coping strategies. | Data were collected by 3 focus group interviews. A semistructured interview guide was constructed. Interviews opened with a question to encourage the informants to narrate a meeting they had had as a nurse in advanced palliative home care with a patient at midlife. To obtain depth in the stories, 2 questions were asked: ‘‘How do you cope with demanding emotions?’’ and ‘‘What kind of support is available to you at work, to handle them?’’ | Fourteen participants, from 3 specialized palliative home care teams, were interviewed in 3 focus groups. Three themes and 9 codes represented the participants’ experiences and coping strategies: experiences of significances: participants felt admiration for the patients, they became invigorated by their encounters with their patients, and they got confirmation that they did well; stress: participants described how they were affected by external influences, psychological stress, and stress-related symptoms; and coping: participants described the various individual-, group-, and organizational-level coping strategies they used in their daily work and the formal and informal support they received. Based on their descriptions, palliative care nursing was stressful for the nurses. They had various coping strategies and significances that helped them care for the families in the most difficult situations | The study further reveals how important the formal support was to the nurses. Studies on experiences of palliative home care by patients in midlife and their spouses are warranted, especially with focus of the home-living children’s situation | Nurse | Interview Study | 17 |
| **9** | Integrated primary palliative care model; facilitators and challenges of primary care/family physicians providing community-based palliative care | 2019 | Atreya, S.; Patil, C.; Kumar, R. | Patients with advanced cancer often suffer from complex symptoms necessitating constant supervision and management. Primary care/family physicians act as an important bridge between the patients in the community and the specialists in the hospital ensuring continuity of care. **(does not really reflect aim / objective, found relevant ans in methods)**  The present paper explored the facilitators and challenges in providing home-based palliative care as perceived by the primary care/family physicians (PCP/FP) | We conducted an exploratory study at Tata Medical Center,  Kolkata between March 2018 and February 2019 to understand  the facilitators and challenges in liaison networking with  PCP/FP as perceived by the latter. The study also explored  PCP/FP’s perceptions of their role and highlighted facilitators  and challenges in delivering primary palliative care within  the community | 62 physicians reported that they were involved in palliative management of at least one cancer patient in the previous year. A significant number of GPs (34%) lacked confidence in providing this care because of patient complexity, inadequate training and insufficient resources. Other barriers included poor communication from specialists and treating teams. Factors facilitating provision of home-based palliative care included their willingness to help palliative care patients, their inclination to train in palliative care and enthusiasm to refer to guidelines while caring for patients. | It is explicit in the paper that resources with respect to information sharing and communication, technical support and training are essential to empower the PCP/FP in providing community-based palliative care. | Primary care physicians and family physicians | Questionnaire study | 6.5 |
| **10** | A qualitative study of home visiting as a palliative care strategy to follow‐up cancer patients by nurses in clinical setting in a developing country | 2019 | Ndiok, Akon; Ncama, Busisiwe | The aims are to examine the effect of home visiting and resources for instituting the programme as a follow‐up strategy in integration of palliative care in daily clinical practice by nurses caring for cancer patients in Nigeria, and on how it can be used to improve care for cancer patients. | This was a qualitative study which employed a focus group discussion with nurse managers and interviews with cancer in‐patients in two teaching hospitals in Nigeria. It utilised an interpretive paradigm to reach the best understanding of the problem. A total of 19 nurse managers who were directly involved with the care of cancer patients and 11 cancer patients participated in the study between July and September 2016. A qualitative content analysis was employed to analyse the data. | Nine major themes were identified in relation to perceived benefits of home visiting services and six in relation to needed resources in instituting the programme. Similar findings emerged from both the focus group discussions and the interviews with patients. Participants agreed that visiting cancer patients would ease many problems commonly encountered by patients following diagnosis of cancer, including psychological, financial, and emotional problems. Needed resources included hospital policies, adequate staff strength, staff commitment and funding. | Viable home visiting requires palliative care teams to carry out the service and hospital policy to direct their activities. Putting this into practice will be in line with World Health Organisation (WHO) advocacy of integrated palliative care for chronic diseases. | Nurse | Focus Group Discussion and Interview Study | 18 |
| **11** | A qualitative evaluation of a home-based palliative care program utilizing community health workers in India | 2019 | Potts, Maryellen; Cartmell, Kathleen; Nemeth, Lynne; Qanungo, Suparna | In India, the need for rural palliative care is increasing with the rising number of people diagnosed with late-stage cancers. Rural areas also have a shortage of trained medical personnel to deliver palliative care. To address these needs, a home-based palliative care program using community health workers (CHWs) to facilitate care delivery was developed to extend the reach of a cancer center's palliative care services outside of Kolkata, India. The research question guiding this qualitative study was, how feasible, useful, and acceptable was this program from the perspectives of the clinical team and CHWs who delivered the intervention? | This qualitative descriptive study used a grounded theory approach and the iterative constant comparative method to collect and analyze data from the key stakeholder interviews. Ten qualitative interviews took place at the Saroj Gupta Cancer Center and Research Institute and were conducted with the CHWs who delivered the home-based palliative care intervention (n = 3) and the clinical team who provided them with training, support, and supervision (n = 7). | Three major themes emerged (a) CHWs' desire and need for more training, (b) the need for tailoring of existing intervention protocols and modifying expectations of stakeholders, and (c) the need for considerations for ensuring program sustainability. | The study provided evidence that the utilization of CHWs to facilitate delivery of palliative care is a feasible model worthy of consideration and further research testing in low-resource settings. | Palliative care clinical team (physicians, nurses, behavioral counselor, and study coordinator)  Community Health Workers (CHWs) | Interview Study | 14 |
| **12** | Experiences of palliative care nurses in providing home-based care for patient with advanced cancer | 2019 | Sijabat, Marlon; Dahlia, Debie; Waluyo, Agung | The main concern of caring for an advanced cancer patient is to fulfill the physical, psychological, social, and spiritual needs to reduce the patient's suffering for a dignified death. The purpose of palliative care is to provide comfort and appropriate palliative care nursing. The courage and dedication to fulfill the complex needs for a dying patient become a unique experience for palliative care nurses. | Such experience was explored by using a descriptive phenomenological approach. Eight palliative care nurses were involved as the participants. The findings from the interview were analyzed by using Colaizzi method. | The analysis resulted six themes: palliative care given after the primary job in hospital, family's assumption of palliative care nurse as a caregiver, palliative care nurse concerned more on patient's physical needs, palliative care nurse more dominant in collaborating to overcome the pain, providing care for imminent death in accordance with predefined standards, and challenges in decision making regarding to palliative care. | It is suggested to improve the services to become a palliative care nurse specialist, to improve knowledge and training of palliative care nurse, and to manage the policy of palliative care nurse in National Health Service system. | Nurses | Interview study | 13 |
| **13** | Burnout in Home Palliative Care: What Is the Role of Coping Strategies? | 2020 | Ercolani, G.; Varani, S.; Peghetti, B.; Franchini, L.; Malerba, M. B.; Messana, R.; Sichi, V.; Pannuti, R.; Pannuti, F. | The study examines psychophysical distress of health-care professionals providing home-based palliative care. The aim is to investigate potential correlations between dimensions of burnout and different coping strategies. | The present study is an observational cross-sectional investigation. The study involved all the home palliative care teams of an Italian nonprofit organization. Of a total of 275 practitioners working for the organization, 207 (75%) decided to participate in the study and complete questionnaires. Questionnaires employed were Maslach Burnout Inventory, General Health Questionnaire 12, Psychophysiological Questionnaire of CBA 2.0, and Coping Orientation to Problems Experienced. Professionals were physicians (50%), nurses (36%), and psychologists (14%). There were no exclusion criteria. Data were processed by SPSS 23 and analyses employed were Spearman ρ, Mann-Whitney U test, and 1-way analysis of variance on ranks. | Among participants, a low number of professionals were emotionally exhausted (11%) or not fulfilled at work (20%), whereas most of them complained of depersonalization symptoms (67%). Emotional exhaustion and depersonalization were found to be associated with avoidance coping strategies, whereas problem-solving and positive attitude were negatively associated with emotional exhaustion and positively with personal accomplishment. Moreover, using avoidance strategies was related to a worse psychological and physical condition. | Findings suggest the need to provide professionals training programs about coping and communication skills tailored to fit the professionals' needs according to their work experience in palliative care and aimed at improving the approach to patients and relatives. | HCPs (physicians, nurses, psychologists) | Questionnaire study | 12.5 |
| **14** | Caring for advanced cancer patients at home during COVID-19 outbreak: burnout and psychological morbidity among palliative care professionals in Italy | 2021 | Varani S, Ostan R, Franchini L, Ercolani G, Pannuti R, Biasco G, Bruera E. | This study aimed to investigate the impact of COVID-19 pandemic on burnout and psychological morbidity among home PCPs in Italy. | One hundred and ninety-eight PC physicians and nurses working in home assistance in Italy were invited to participate. The results obtained by the investigation conducted during the COVID-19 emergency (COVID2020) were compared with data collected in 2016 in the same setting (BURNOUT2016). The questionnaires (socio-demographics, Maslach Burnout Inventory and General Health Questionnaire-12) were the same for both the surveys. The PCPs participating in COVID2020 survey (n ¼ 145) were mostly the same (70%) who participated in the BURNOUT2016 study (n ¼ 179). | One hundred and forty-five PCPs participated in the study (response rate 73.2%). During the COVID-19 emergency, home PCPs presented a lower burnout frequency (P <.001) and higher level of personal accomplishment than in 2016 (P ¼ .047). Conversely, the risk for psychological morbidity was significantly higher during the pandemic (P < .001). | In the age of COVID-19, the awareness of being at the forefront of containing the pandemic along with the sense of responsibility toward their high-risk patients may arouse PCPs’ psychological distress, but, on the other hand, this condition may improve their sense of professional satisfaction and personal accomplishment | Doctors and Nurses | Questionnaire study | 11 |
| **15** | Talking together in rural palliative care: a qualitative study of interprofessional collaboration in Norway | 2022 | Johansen, May-Lill; Ervik, Bente | To explore how rural health professionals experience local and regional collaboration on patients in need of palliative care. | This was a qualitative focus group and interview study in rural Northern Norway involving 52 primary care health professionals including district nurses, general practitioners, oncology nurses, physiotherapists, and occupational therapists. Five uniprofessional focus group discussions were followed by five interprofessional discussions and six individual interviews. Transcripts were analysed thematically. | Talking together” was perceived as the optimal form of collaboration, both within primary care and with specialists. Nurses and GPs had similar perceptions of their worstcase scenario in primary palliative care: the sudden arrival after working hours of a sick patient about whom they lacked information. These situations could be the result of a short notice transfer from secondary care or an emergency presentation after a crisis in patient management locally, the latter often resulting in a hospital admission. Participants missed timely and detailed discharge letters and in complex cases a telephone call or conference. Locally, colocation was perceived as advantageous for crucial communication, mutual support, and knowledge about each other’s competencies and work schedule. Because local health professionals belonged to diferent units within the primary health care organisation, in some places they had limited knowledge about each other’s roles and skill sets. | Lack of communication, both locally and between specialist and primary care, was a key factor in the worst case patient scenarios for GPs and nurses working in primary palliative care in rural Northern Norway. Co location of primary care professionals promoted local collaboration and should be encouraged. Hospital discharge planning should involve the receiving primary care professionals. | HCPs (district nurses, general practitioners, oncology nurses, physiotherapists, and occupational therapists) | Focus Group and Interview Study | 19 |
| **16** | Delivering end‐of‐life care for patients with cancer at home: Interviews exploring the views and experiences of general practitioners | 2022 | Wyatt, Kelly; Bastaki, Hamad; Davies, Nathan | The aim of this study was to explore general practitioners experiences of providing end‐of‐life care for people with cancer in the home setting and their perceptions of confidence in this role as well as understanding implications this has on policy design. | A qualitative study using semi-structured interviews, guided by an interview schedule, developed from the literature and discussions among the research team. Interviews were analysed using thematic analysis (Braun & Clarke., 2006), guided by the data adopting an explorative and inductive approach. | Five main themes were constructed: (a) the subjective nature of defining palliative and end‐of‐life care; (b) importance of communication and managing expectations; (c) complexity in prescribing; (d) challenging nature of delivering end‐of‐life care; (e) the unclear role of primary care in palliative care. General practitioners viewed end‐of‐life care as challenging; specific difficulties surrounded communication and prescribing. These challenges coupled with a poorly defined role created a spread in perceived confidence. Experience and exposure were seen as enabling confidence. Specialist palliative care service expansion had important implications on deskilling of essential competencies and reducing confidence levels in general practitioners. This feeds into a complex cycle of causation, leading to further delegation of care. | GPs viewed end-of-life care as challenging, specific difficulties included communication and pain-management. These challenges together with lacking role clarity led to ranging confidence levels, with experience and exposure seen as enablers of confidence. Consideration needs to be taken with the expansion of specialist palliative services and the implication this has on deskilling essential competencies, reducing confidence levels in GPs and in a cycle of causation, leading to further delegation of care. | Doctors | Interview Study | 26 |
| **17** | Timely Identification of Patients With Cancer and Family Caregivers in Need of End-of-Life Discussions by Home-Visit Nurses in Japan: A Qualitative Descriptive Study | 2023 | Asaumi, K.; Oki, M.; Murakami, Y. | To identify the time at which patients with cancer and their caregivers need EOL discussions, we explored when home-visit nurses start EOL discussions. | We interviewed 23 home-visit nurses and analyzed the data using qualitative content analysis | Three themes were derived from the analysis. Participants identified the timing of EOL discussions as being sensitive to patients’ changing health and care needs (increases in patient’s total pain), changes in the family caregiver’s physical or mental condition through daily care (increases in family caregiver distress), and the EOL process that patients follow (trajectory of disease) | Developing a tool or in-service educational program that will enable inexperienced or new graduate home-visit nurses to implement EOL discussions at appropriate times is necessary | Nurses | Interview study | 21 |
| **18** | Who is the key worker in palliative home care? Views of patients, relatives and primary care professionals | 2011 | Brogaard, Trine; Jensen, Anders Bonde; Sokolowski, Ineta; Olesen, Frede; Neergaard, Mette Asbjorn | Palliative home care involves coordination of care between the professionals involved. The NICE guideline on supportive and palliative care (UK) recommends that teams, regardless of their base, should promote continuity for patients. This may involve nomination of a coordinating “key worker”. This study aimed to explore who acts as key worker and who ought to take on this role in the views of patients, relatives, and primary care professionals. Furthermore, it aimed to explore the level of agreement on this issue between study participants. | Actual key worker as valued by patients, relatives, and primary care professionals; ideal key worker as valued by patients and relatives. | Patients, relatives, GPs, and CNs most often saw themselves as having been the key worker. When asked about the ideal key worker, most patients (29%; 95%CI: 18;42) and relatives (32%; 95%CI: 22;45) pointed to the GP. Using patients’ views as reference, we found very limited agreement with relatives (47.7%; k0.05), with GPs (30.4%; k0.01) and with CNs (25.0%; k0.04). Agreement between patients and relatives on the identity of the ideal key worker was of a similar dimension (29.6%; k  0.11). | Poor agreement between patients, relatives, and professionals on actual and ideal key worker emphasizes the need for matching expectations and clear communication about task distribution in palliative home care. | Terminally ill cancer patients, their relatives, general practitioners (GPs), and community nurses (CNs) | Mixed - Interview and questionnaire study | COREQ: 8  MERSQI:  8.5 |
| **19** | Rural palliative care to support dying at home can be realised; experiences of family members and nurses with a new model of care | 2019 | Spelten, E.; Timmis, J.; Heald, S.; Duijts, S. F. A. | While 60%‐70% of people would prefer to die at home, only 14% do so. Families in a rural environment feel particularly unsupported in fulfilling this last wish of their loved one, which reflects the general shortage of health care workforce and resource allocation to institutions. The aim of this study was to describe the experience of families and nurses with extended rural palliative care to support dying at home. | Semistructured interviews were conducted with family members and nurses. Process data were included to describe the frequency and nature of contacts. The results were analysed using descriptive analysis. | All patients in the project died at home. The families were very positive about the extended palliative care; it increased their familiarity with dying, and had a positive impact on bereavement. The nurses were equally positive, but also commented on the need to debrief and on the heavy emotional toll the work takes. | Rural care support for dying at home can be realised. This study has demonstrated the positive impact of an end‐of‐life service, while at the same time pointing to concerns of the nursing staff on the suitability of the model of care. Health care workers and communities alike need to be educated and have conversations on end‐of‐life care. | Family members and Nurses | Interview Study | 16 |
| **20** | Barriers to home‐based palliative care in people with cancer: A qualitative study of the perspective of caregivers | 2020 | Hassankhani, Hadi; Rahmani, Azad; Best, Amy; Taleghani, Fariba; Sanaat, Zohreh; Dehghannezhad, Javad | To investigate the barriers to home-based palliative care for cancer patients from professional caregivers' experiences. | This is a descriptive-qualitative study carried out in the community-based care. Twenty-three participants took part in this study. Data were collected through semi-structured interviews. | Data analysis led to the identification of three category of barriers including the lack of instructions (the lack of clinical practice guidelines, the ambiguity of tariffs and the lack of insurance coverage), family desperation (family views of prognosis, distrust and poverty) and lack of professionalism (limited knowledge, the use of amateur nurses and siloed care). Developing a care protocol and providing resources support contribute to the development of home-based palliative care. Moreover, the education of families and training courses for nurses must be fostered. |  | Managers of community-based care, nurses with the experience of providing home care to cancer patients, an oncologist, a pain specialist, nursing doctors and the nursing manager | Interview Study | 17 |
